# Association Between Length of Only-Child Period During Early Childhood and Overweight at Age 8—A Population-Based Longitudinal Study in Japan

**DOI:** 10.3389/fped.2022.782940

**Published:** 2022-06-14

**Authors:** Aomi Katagiri, Nobutoshi Nawa, Takeo Fujiwara

**Affiliations:** ^1^Department of Global Health Promotion, Tokyo Medical and Dental University, Tokyo, Japan; ^2^Department of Medical Education Research and Development, Tokyo Medical and Dental University, Tokyo, Japan

**Keywords:** only-child period, birth order, childhood overweight, longitudinal study, Japan

## Abstract

**Introduction:**

Prior studies have shown that children who are the only child are more likely to be overweight compared to their peers with siblings, regardless of whether they are the oldest, in the middle, or youngest. The study objective was to clarify whether there is an association between the length of the only-child period and the risk of overweight in firstborns who experienced an only-child period during early childhood before their siblings were born.

**Methods:**

A total of 7,576 first-born boys and 7,229 first-born girls were examined from a nationwide longitudinal survey in Japan. The length of the only-child period was determined by “birth interval”; i.e., the interval between the birth of the index child and the birth of the second child. It was categorized as short (<1.5 years), moderate (between 1.5 and 4 years), long (between 4 and 8 years), and only-child (the second baby was not born for 8 years). Overweight was defined as body mass index (BMI) z-score 1 standard deviation or more at age 8. Logistic regression was used to examine the association between length of only-child period and childhood overweight, adjusting for covariates.

**Results:**

Moderate birth interval was inversely associated with being overweight in comparison with only-child in both boys (odds ratio (OR): 0.83, 95% CI, 0.72–0.96) and girls (OR: 0.75, 95% CI, 0.63–0.88). Long birth interval also showed inverse association in boys (OR: 0.78, 95% CI, 0.62–0.97), and marginal inverse association in girls (OR: 0.80, 95% CI, 0.62–1.04).

**Conclusion:**

First-born children who experienced short birth intervals did not show a different overweight risk from only-child. First-born children who experienced 1.5–8 years of the birth interval had a lower risk of childhood overweight compared with only-child.

## Introduction

Childhood obesity and overweight remain major public health problems, given their link to lifelong influence on both physical and mental health in the future (1–3). Risk factors for overweight during childhood include demographic factors such as low socioeconomic status (4), single-parenthood (2), and grandparent cohabitation (5, 6), and also early lifestyle habits such as high-calorie intake and excessive screen time (7). In addition to these factors, birth order has been studied in relation to overweight during childhood. Prior studies have shown that children who are the only child are more likely to be overweight compared to their peers with siblings, regardless of whether they are the oldest, in the middle, or youngest (8–11).

The elevated risk of being overweight among children who are the only child can be explained by the unique environments in which they grow up. Previous studies have shown that those who are the only child received more attention from their parents (12), and in particular, maternal involvement in eating behavior may have led to higher intake and overweight in those who are the only child (12). It was also reported that children who were the only child were probably more likely to be provided with a larger amount of food compared to those with siblings (13). Conversely, children who have siblings are required to share resources including food with their peers (10, 13). Less physical exercise due to having no siblings may also contribute to childhood overweight in those who are the only child (8, 14, 15).

However, no study to date has focused on the difference in overweight risk among first-born children (i.e., index children) who have siblings with respect to the birth interval between the index and subsequent child. The hypothesis is that when the first-born child has a shorter age gap than the second-born sibling, the risk of being overweight will be lower because the index child will soon face the birth of their sibling and may not receive enough resources including food (10, 13). Moreover, most studies on birth intervals have only examined the outcome of the subsequent child, and not the index child (16, 17). Hence, the association between birth intervals and overweight in childhood among index children has not been sufficiently investigated.

To that end, this study aims to assess the association between birth intervals and overweight in childhood among index children using data from a nationwide longitudinal survey conducted in Japan from 2001 to 2009.

## Methods

### Sample

Data from the Longitudinal Survey of Newborns in the 21st Century, conducted by the Ministry of Health, Labor and Welfare in Japan between 2001 and 2009 (18) was used. According to the national vital registration system of Japan, all babies born between 10th and 17th January and 10th and 17th July in 2001 were identified for the survey (*n* = 53,575) (18). A previous profile paper on this cohort indicates that is a representative sample of Japanese children (18). Questionnaires were sent *via* postal mail when children were at the age of 0.5, 1.5, 2.5, 3.5, 4.5, 5.5, 7, and 8 years old. Return of the questionnaire to the Ministry of Health, Labor and Welfare was considered as the consent of participation in the study, as in previous studies (19, 20), resulting in 47,015 participants (87.8%) for the first wave at the age of 0.5 years old. The response rate for eighth survey was 76.9% (*n* = 36,151). All data were reported by guardians (i.e., parents or grandparents). When participants did not reply for two consecutive years, they were considered a dropout.

**Figure 1:**
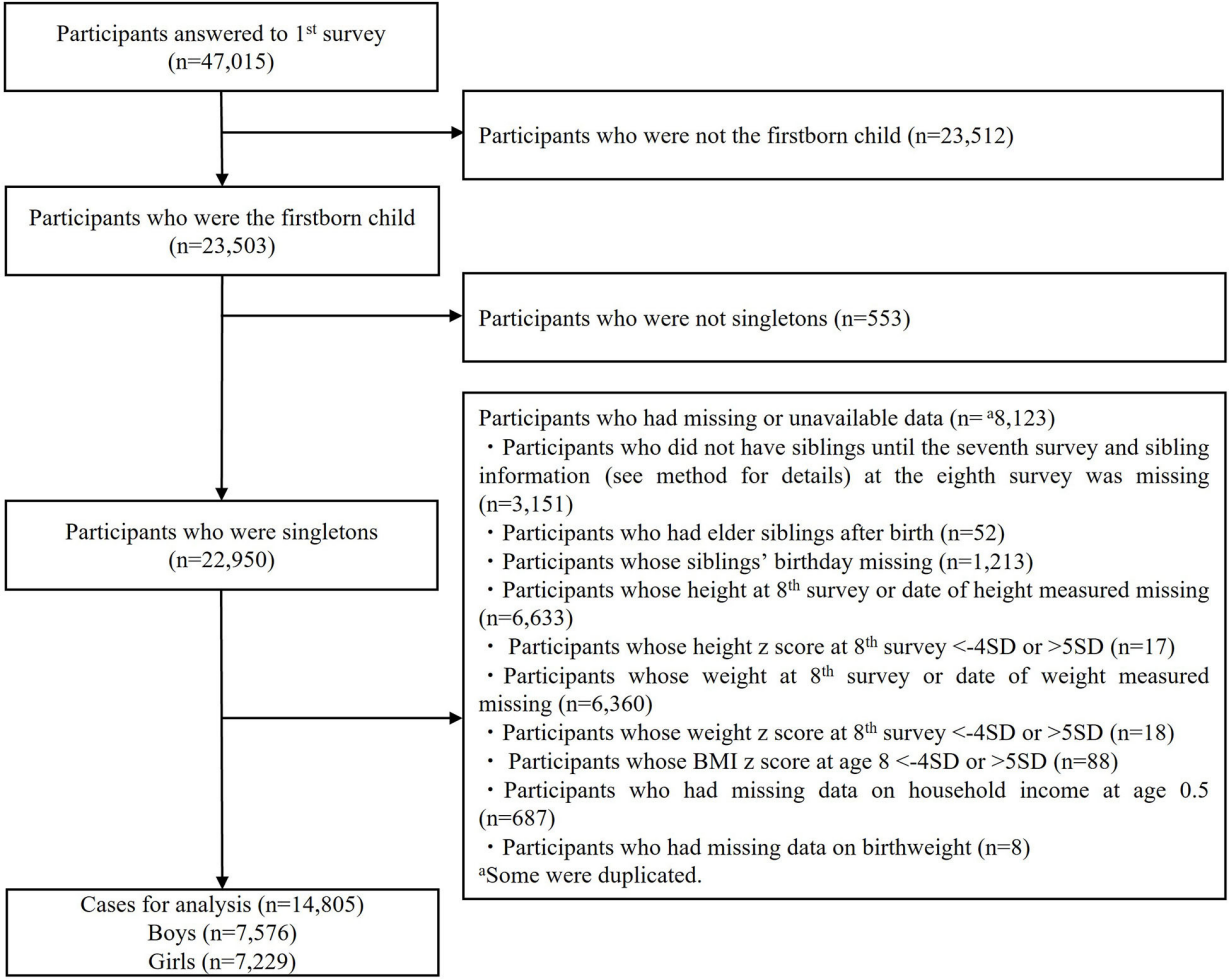
Enrollment of study participants.

Exclusion criteria were participants who were not the first-born child (*n* = 23,512); who were not singletons (*n* = 553); and who did not have siblings until the seventh survey and sibling information at the eighth survey was missing (*n* = 3,151); who had elder siblings after birth (*n* = 52); whose siblings' birthday was missing (*n* = 1,213); whose height at the eighth survey or date of height measured was missing (*n* = 6,633); whose height *z*-score at the eighth survey was <-4 SD or >5 SD (*n* = 17); whose weight at the eighth survey or date of weight measured was missing (*n* = 6,360); whose weight z-score at the eighth survey was <-4 SD or >5 SD (*n* = 18); whose body mass index (BMI) *z*-score at the eighth survey was <-4 SD or >5 SD (*n* = 88); and whose information on continuous covariates, such as household income at the first wave (*n* = 687) and birth weight (*n* = 8), was missing. This resulted in an analytic sample size of 7,576 boys and 7,229 girls (Figure 1). *Z*-score was unable to be calculated when there was missing data on either height, weight, or concrete age at the eighth survey. Participants whose z-score was <-4 SD or >5 SD were excluded because they were regarded as outliers in accordance with methods commonly applied in epidemiological studies, as suggested by the Center for Disease and Control (21). In sensitivity analysis, participants whose BMI *z*-score at birth or BMI *z*-score at second wave was not available due to missing information on either height, weight, or date of measurement (*n* = 1,406), and participants whose BMI z-score was regarded as outliers (under −4 SD or over 5 SD) (*n* = 123) were excluded, resulting in 6,779 boys and 6,507 girls. This study was approved by the ethics committee at Tokyo Medical and Dental University (M2019-066).

### Measurements

#### Exposure

Birth order and singleton status were determined from the first survey. The number of siblings and birth intervals were determined from the first to eighth wave, namely, ages of 0.5–8 years old. Birth intervals were calculated by subtracting the index child's birthday from the subsequent child's birthday using data from the first to the fifth wave. Since only information on year and month was available for the subsequent child's birth, the birth interval was calculated assuming that the birth date was the 15th.

#### Outcome

Overweight at the age of 8 years old was used as the outcome of interest. Overweight was determined as BMI z-score 1 SD or more according to the WHO criteria (22). BMI was calculated as weight (kg) divided by the square of height (m). Height and weight were reported by guardians in every questionnaire. The plausibility of height and weight reported by caregivers has been documented in previous studies (19, 23).

### Covariates

Covariates were selected according to previous studies conducted among birth order and overweight in childhood (4, 10, 24). Covariates included household income at the age of 0.5 years old, birth weight, mother's age at birth, mother's education level, mother's smoking status at the age of 0.5 years old, and father cohabitation at the age of 0.5 years old because such factors are related with both timing of siblings' birth and childhood overweight. We included maternal smoking status as a covariate because smoking is associated with lower socioeconomic status (25). Father cohabitation was included as a covariate because the survey did not have questions on marital status, and single parenthood may decrease the possibility of future siblings and is known to lead to childhood overweight (26).

The other factors were preterm birth, duration of breastfeeding, rapid growth during infancy, absence of exercise habits, skipping breakfast, excessive screen time, irregular sleeping hours, unhealthy dinner habits, and grandparent cohabitation at the age of 8 years. These variables were included based on previous research (6, 24, 27–29).

Equivalized household income at the age of 0.5 years old was calculated as total household income (i.e., the sum of the mother's and father's income), divided by the square root of the number of people in the household, based on responses from the first wave (30). Household income at the age of 0.5 years old was scaled so that the effect of a one-unit change in income can be interpreted as the effect of a one million JPY change in income. Birth weight was collected from the birth record and was scaled so that the effect of a one-unit change in weight can be interpreted as the effect of a 0.1-kg change in weight. The mother's age at birth was calculated by subtracting the mother's birthday from the first-born child's birthday collected from the birth record and the first wave. The mother's age at birth was scaled so that the effect of a one-unit change in age can be interpreted as the effect of a 10-year change in age. The education level of the mother was collected in the second wave and categorized as junior high school, high school, vocational school, and higher education. Preterm birth was defined as <37 gestational weeks according to the birth record (31). Maternal smoking status was collected in the first wave and categorized as smoking and no smoking. Duration of breastfeeding was determined as None, 1–2 months, 3–5 months, and 6 or more months according to the question at the first wave. Rapid growth during infancy was determined as gaining more than 0.67 SD of weight from age 0.5 years to age 1.5 years (32). Exercise habits were determined as yes if the participants joined sports clubs or lessons such as gymnastics, swimming, baseball, soccer, tennis, martial arts, or dance, and no if they did not join any of these activities at the age of 8 years old. This is in accordance with previous research on pediatric overweight that defined physical activities as for at least 60 min outside of school education throughout a week (27). Skipping breakfast was asked with a yes-no question at the eighth wave. Excessive screen time was determined as two or more hours of watching TV or playing computer games on either weekdays or weekends at the age of 8 years old which leads to a high level of sedentary time (33). Irregular sleeping hours were determined if the child had irregular bedtime or irregular time of getting up on either weekdays or weekends, which was asked with a yes-no question at the age of 8 years old, in accordance with previous research that report an association between high variety in sleep patterns and pediatric overweight (29). Unhealthy dinner habits were determined by frequent or occasional consumption of instant food and pre-cooked food, using food delivery, or eating out on weekdays, which was asked at the age of 8 years old, as previous research reports the association with fast food consumption and childhood overweight (27). Grandparent cohabitation at the age of 8 years old was determined as the cohabitation of one or more grandparents living together.

### Statistical Analysis

Statistical analysis was conducted using a logistic regression model to estimate the odds ratio for overweight at the age of 8 years old. In sensitivity analysis, samples were restricted to those whose mother's age was under 35 years old (*n* = 13,497). A separate analysis was conducted among boys and girls as in previous research on childhood obesity using the same population (34). All categorical variables were converted into dummy variables and put into regression models. When a variable contains a missing value, a dummy variable was also created to indicate whether the value of the variable is missing or not. Sensitivity analysis was also conducted including BMI z-score at birth and change of BMI z-score from birth to the second wave (age 1.5) to take into account the presence of overweight in early childhood and changes in BMI z-score during early childhood according to previous research (32). All analyses were performed with STATA SE statistical package, version 14 (StataCorporation. Stata statistical software, release 14. College Station, TX: StataCorporation LP; 2015).

## Results

The percentage of boys who were overweight at the age of 8 years old was 21.5, 19.9, 17.1, and 16.2% among only-child, index child with a birth interval shorter than 1.5 years, index child with a birth interval between 1.5 years and 4 years, and index child with a birth interval between 4 years and 8 years, respectively (Table 2). Among girls, the percentages were 15.4%, 14.2%, 11.2%, and 11.9%, respectively (Table 3). The average household income at the age of 0.5 years old was lowest in first-born children who experienced an age gap shorter than 1.5 years among both boys and girls (Tables 1–3). Mother's age at birth was highest in only-child among both boys and girls (Tables 1–3). Father residence at the age of 0.5 years old was more likely to be seen among children who had siblings in both boys and girls (Tables 1–3). First-born boys who experienced birth intervals between 1.5 years and 4 years had 18% lower odds (OR 0.82, 95% CI, 0.71–0.94) of being overweight compared to only-child after adjusting for potential confounders (Table 4, Model 1). Moreover, boys who experienced birth intervals between 4 years and 8 years had 25% lower odds (OR 0.75, 95% CI, 0.61–0.93) of being overweight as well, while the odds ratio (OR) for boys who experienced birth intervals shorter than 1.5 years was 0.93 (95% CI, 0.70–1.23), compared to an only child. First-born girls who experienced birth intervals between 1.5 and 4 years had 27% lower odds (OR 0.73, 95% CI, 0.62–0.86) of being overweight compared to an only child after adjusting for potential confounders (Table 5, Model 1). However, the OR for girls who experienced birth intervals between 4 and 8 years was 0.79 (95% CI, 0.61–1.01), and the OR for those who experienced birth intervals shorter than 1.5 years was 0.89 (95% CI, 0.64–1.25), compared to an only child.

**Table 1:** Characteristics of study participants from the Longitudinal Survey of Newborns in the 21st Century, 2001–2009 (total *n* = 14,805).

		**Only children**	**Age gap shorter** **than 1.5 years**	**Age gap 1.5 years to shorter than 4 years**	**Age gap 4–8 years**
**Variable**		**No (%) or mean (SD)**	**No (%) or mean (SD)**	**No (%) or mean (SD)**	**No (%) or mean (SD)**
Number of participants		4,380 (29.6)	697 (4.7)	8,065 (54.5)	1,663 (11.2)
Overweight^a^	Yes	813 (18.6)	120 (17.2)	1,150 (14.3)	234 (14.1)
	No	3,567 (81.4)	577 (82.8)	6,915 (85.7)	1,429 (85.9)
Household income at age 0.5, million JPY	Mean, SD	3.9 (2.6)	3.5 (2.0)	3.82 (2.2)	3.9 (3.1)
Birth weight	<2,500 g	408 (9.3)	46 (6.6)	593 (7.4)	142 (8.5)
	≧2,500 g	3,972 (90.7)	651 (93.4)	7,472 (92.7)	1,521 (91.5)
Mother's age at birth, years	Mean, SD	30.8 (4.9)	27.6 (4.2)	28.3 (3.6)	28.5 (3.6)
	<25	494 (11.3)	198 (28.4)	1,336 (16.6)	244 (14.7)
	25–29	1,500 (34.3)	313 (44.9)	4,311 (53.5)	874 (52.6)
	30–34	1,501 (34.3)	149 (21.4)	2,096 (26.0)	481 (28.9)
	35–39	734 (16.8)	35 (5.0)	313 (3.9)	64 (3.9)
	≧40	151 (3.5)	2 (0.3)	9 (0.1)	0 (0.0)
Mother's education	Junior high school	155 (3.5)	29 (4.2)	160 (2.0)	24 (1.4)
	High school	1,592 (36.4)	287 (41.2)	2,582 (32.0)	493 (29.7)
	Vocational school	1,782 (40.7)	285 (40.9)	3,800 (47.1)	829 (49.9)
	Higher education	751 (17.2)	80 (11.5)	1,415 (17.5)	294 (17.7)
	Others	6 (0.1)	4 (0.6)	7 (0.1)	1 (0.1)
	Missing	94 (2.2)	12 (1.7)	101 (1.3)	22 (1.3)
Mother's smoking status at age 0.5	No	3,660 (83.6)	589 (84.5)	7,131 (88.4)	1,437 (86.4)
	Yes	692 (15.8)	105 (15.1)	915 (11.4)	222 (13.4)
	Missing	28 (0.6)	3 (0.4)	19 (0.2)	4 (0.2)
Father residence at age 0.5	Yes	4,152 (90.7)	688 (98.7)	7,999 (99.2)	24 (1.4)
	No	228 (9.3)	9 (1.3)	66 (0.8)	1,639 (98.6)
Gestational period, weeks	22–36	229 (5.2)	19 (2.7)	259 (3.2)	53 (3.2)
	37–41	4,093 (93.5)	668 (95.8)	7,705 (95.5)	1,582 (95.1)
	≧42	58 (1.3)	10 (1.4)	101 (1.3)	28 (1.7)
Duration of breastfeeding	None	391 (8.9)	39 (5.6)	334 (4.1)	56 (3.4)
	1–2 months	948 (21.6)	217 (31.1)	1,175 (14.6)	268 (16.1)
	3–5 months	907 (20.7)	220 (31.6)	1,540 (19.1)	361 (21.7)
	6 or more months	2,095 (47.8)	215 (30.9)	4,917 (61.0)	958 (57.6)
	Missing	39 (0.9)	6 (0.9)	99 (1.2)	20 (1.2)
Rapid growth during early childhood^b^	No	2,262 (51.6)	346 (49.6)	4,421 (54.8)	924 (55.6)
	Yes	1,727 (39.4)	268 (38.5)	3,069 (38.1)	607 (36.5)
	Missing	391 (8.9)	83 (11.9)	575 (7.1)	132 (7.9)
Exercise habit at age 8	Yes	2,425 (55.4)	354 (50.8)	4,635 (57.5)	992 (59.7)
	No	1,955 (44.6)	343 (49.2)	3,430 (42.5)	671 (40.4)
Breakfast consumption at age 8	Yes	4,132 (94.3)	665 (95.4)	7,722 (95.8)	1,589 (95.6)
	No	146 (3.3)	21 (3.0)	204 (2.5)	50 (3.0)
	Missing	102 (2.3)	11 (1.6)	139 (1.7)	24 (1.4)
Excessive screen time at age 8^c^	No	1,333 (30.4)	222 (31.9)	2,704 (33.5)	492 (29.6)
	Yes	3,039 (69.4)	475 (68.2)	5,349 (66.3)	1,168 (70.2)
	Missing	8 (0.2)	0 (0.0)	12 (0.2)	3 (0.2)
Irregular bedtime at age 8	No	4,223 (96.4)	684 (98.1)	7,925 (98.3)	1,621 (97.5)
	Yes	151 (3.5)	12 (1.7)	128 (1.6)	39 (2.4)
	Missing	6 (0.1)	1 (0.1)	12 (0.2)	3 (0.2)
Healthy dinner habits at age 8^d^	Yes	1,208 (27.6)	197 (28.3)	2,721 (33.7)	555 (33.4)
	No	3,159 (72.1)	497 (71.3)	5,316 (65.9)	1,102 (66.3)
	Missing	13 (0.3)	3 (0.4)	28 (0.4)	6 (0.4)
Grandparent cohabitation at age 8	No	3,270 (74.7)	528 (75.8)	6,313 (78.3)	1,349 (81.1)
	Yes	1,110 (25.3)	169 (24.3)	1,752 (21.7)	314 (18.9)
BMI at birth	Mean, SD	12.5 (1.1)	12.6 (1.1)	12.5 (1.1)	12.5 (1.1)
	Unavailable	63 (1.4)	7 (1.0)	54 (0.7)	22 (1.3)
BMI at age 1.5	Mean, SD	16.3 (1.4)	16.4 (1.4)	16.3 (1.3)	16.3 (1.4)
	Unavailable	429 (9.8)	102 (14.6)	706 (8.8)	151 (9.1)
Father's age at first birth, years	Mean, SD	33.0 (6.1)	30.0 (5.5)	30.2 (4.6)	30.3 (4.4)
	<25	310 (7.1)	113 (16.2)	847 (10.5)	154 (9.3)
	25–29	1,084 (24.8)	278 (39.9)	3,451 (42.8)	650 (39.1)
	30–34	1,407 (32.1)	180 (25.8)	2,594 (32.2)	627 (37.7)
	35–39	926 (21.1)	85 (12.2)	907 (11.3)	185 (1.1)
	≧40	526 (12.0)	32 (4.6)	233 (2.9)	36 (2.2)
	Missing	127 (2.9)	9 (1.3)	33 (0.4)	11 (0.7)
Father's education	Junior high school	239 (5.5)	55 (7.9)	373 (4.6)	69 (4.2)
	High school	1,485 (33.9)	303 (43.5)	2,903 (36.0)	563 (33.9)
	Vocational school	702 (16.0)	121 (17.4)	1,442 (17.9)	296 (17.8)
	Higher education	1,731 (39.5)	200 (28.7)	3,225 (40.0)	708 (42.6)
	Others	4 (0.1)	0 (0.0)	3 (0.04)	3 (0.2)
	Missing	219 (5.0)	18 (2.6)	119 (1.5)	24 (1.4)
Father's smoking status at age 0.5	No	1,665 (38.0)	212 (30.4)	3,316 (41.1)	673 (40.5)
	Yes	2,512 (57.4)	477 (68.4)	4,698 (58.3)	969 (58.3)
	Missing	203 (4.6)	8 (1.2)	51 (0.6)	21 (1.3)
Residential area	20 designated cities	1,234 (28.2)	130 (18.7)	1,726 (21.4)	407 (24.5)
	Other cities	2,479 (56.6)	393 (56.4)	4,845 (60.1)	1,000 (60.1)
	Rural	667 (15.2)	174 (25.0)	1,494 (18.5)	256 (15.4)

*SD, standard deviation.*

a *Overweight was determined as BMI z-score 1 SD or more according to the WHO criteria at the age of 8 years old (22).*

b *Rapid growth during infancy was determined as gaining more than 0.67 SD of weight from age 0.5 years to age 1.5 years (32).*

c *Excessive screen time was determined as 2 or more h of watching TV or playing computer games on either weekdays or weekends at the age of 8 years old (33).*

d *Unhealthy dinner habits were determined by frequent or occasional consumption of instant food and precooked food, using food delivery, or eating out on weekdays, which was asked at the age of 8 years old*.

**Table 2:** Characteristics of study participants from the Longitudinal Survey of Newborns in the 21st Century, 2001–2009 (boys *n* = 7,576).

		**Only children**	**Age gap shorter than 1.5 years**	**Age gap 1.5 years to shorter than 4 years**	**Age gap 4–8 years**
**Variable**		**No (%) or mean (SD)**	**No (%) or mean (SD)**	**No (%) or mean (SD)**	**No (%) or mean (SD)**
Number of participants		2,247 (29.7)	367 (4.8)	4,115 (54.3)	847 (11.2)
Overweight^a^	Yes	484 (21.5)	73 (19.9)	709 (17.1)	137 (16.2)
	No	1,763 (78.5)	294 (80.1)	3,406 (82.9)	710 (83.8)
Household income at age 0.5, million JPY	Mean, SD	4.0 (2.8)	3.4 (1.9)	3.8 (2.2)	3.9 (1.9)
Birth weight	<2,500 g	185 (8.2)	20 (5.5)	264 (6.4)	61 (7.2)
	≧2,500 g	2,062 (91.8)	347 (94.6)	3,851 (93.6)	786 (92.8)
Mother's age at birth, years	Mean, SD	30.8 (5.0)	27.3 (4.2)	28.3 (3.6)	28.5 (3.4)
	<25	253 (11.3)	118 (32.2)	689 (16.7)	111 (13.1)
	25**–**29	771 (34.3)	157 (42.8)	2,219 (53.9)	465 (54.9)
	30−34	757 (33.7)	75 (20.4)	1,042 (25.3)	243 (28.7)
	35**–**39	375 (16.7)	17 (4.6)	161 (3.9)	28 (3.3)
	≧40	91 (4.1)	0 (0.0)	4 (0.1)	0 (0.0)
Mother's education	Junior high school	87 (3.9)	15 (4.1)	78 (1.9)	14 (1.7)
	High school	831 (37.0)	155 (42.2)	1,282 (31.2)	249 (29.4)
	Vocational school	929 (41.3)	146 (39.8)	1,979 (48.1)	415 (49.0)
	Higher education	351 (15.6)	41 (11.2)	719 (17.5)	150 (17.7)
	Others	4 (0.2)	2 (0.5)	4 (0.1)	1 (0.1)
	Missing	45 (2.0)	8 (2.2)	53 (1.3)	18 (2.1)
Mother's smoking status at age 0.5	No	1,868 (83.1)	315 (85.8)	3,666 (89.1)	741 (87.5)
	Yes	361 (16.1)	51 (13.9)	439 (10.7)	105 (12.4)
	Missing	18 (0.8)	1 (0.3)	10 (0.2)	1 (0.1)
Father residence at age 0.5	Yes	2,131 (94.8)	362 (98.6)	4,083 (99.2)	836 (98.7)
	No	116 (5.2)	5 (1.4)	32 (0.8)	11 (1.3)
Gestational period, weeks	22**–**36	135 (6.0)	8 (2.2)	157 (3.8)	31 (3.7)
	37**–**41	2,083 (92.7)	353 (96.2)	3,903 (94.9)	804 (94.9)
	≧42	29 (1.3)	6 (1.6)	55 (1.3)	12 (1.4)
Duration of breastfeeding	None	204 (9.1)	21 (5.7)	176 (4.3)	32 (3.8)
	1**–**2 months	486 (21.6)	114 (31.1)	611 (14.9)	127 (15.0)
	3**–**5 months	437 (19.5)	112 (30.5)	739 (18.0)	183 (21.6)
	6 or more months	1,096 (48.8)	117 (31.9)	2,535 (61.6)	498 (58.8)
	Missing	24 (1.1)	3 (0.8)	54 (1.3)	7 (0.8)
Rapid growth during early childhood^b^	No	1,166 (51.9)	185 (50.4)	2,264 (55.0)	464 (54.8)
	Yes	891 (39.7)	141 (38.4)	1,544 (37.5)	308 (36.4)
	Missing	190 (8.5)	41 (11.2)	307 (7.5)	75 (8.9)
Exercise habit at age 8	Yes	1,361 (60.6)	209 (57.0)	2,688 (65.3)	575 (67.9)
	No	886 (39.4)	158 (43.1)	1427 (34.7)	272 (32.1)
Breakfast consumption at age 8	Yes	2,112 (94.0)	349 (95.1)	3,936 (95.7)	814 (96.1)
	No	87 (3.9)	10 (2.7)	110 (2.7)	21 (2.5)
	Missing	48 (2.1)	8 (2.2)	69 (1.7)	12 (1.4)
Excessive screen time at age 8^c^	No	660 (29.4)	102 (27.8)	1,315 (32.0)	261 (30.8)
	Yes	1,583 (70.5)	265 (72.2)	2,793 (67.9)	584 (69.0)
	Missing	4 (0.2)	0 (0.0)	7 (0.2)	2 (0.2)
Irregular bedtime at age 8	No	2,158 (96.0)	364 (99.2)	4,047 (98.4)	830 (98.0)
	Yes	87 (3.9)	2 (0.5)	60 (1.5)	15 (1.8)
	Missing	2 (0.1)	1 (0.3)	8 (0.2)	2 (0.2)
Healthy dinner habits at age 8^d^	Yes	597 (26.6)	93 (25.3)	1,368 (33.2)	272 (32.1)
	No	1,644 (73.2)	273 (74.4)	2740 (66.6)	573 (67.7)
	Missing	6 (0.3)	1 (0.3)	7 (0.2)	2 (0.2)
Grandparent cohabitation at age 8	No	1,678 (74.7)	268 (73.0)	3,190 (77.5)	689 (81.4)
	Yes	569 (25.3)	99 (27.0)	925 (22.5)	158 (18.7)
BMI at birth	Mean, SD	12.5 (1.1)	12.6 (1.1)	12.6 (1.1)	12.6 (1.1)
	Unavailable	38 (1.7)	4 (1.1)	28 (0.7)	9 (1.1)
BMI at age 1.5	Mean, SD	16.5 (1.3)	16.6 (1.4)	16.5 (1.3)	16.4 (1.4)
	Unavailable	211 (9.4)	51 (13.9)	382 (9.3)	81 (9.6)
Father's age at first birth, years	Mean, SD	33.2 (6.3)	29.7 (18.4)	30.1 (4.4)	30.5 (4.4)
	<25	150 (6.7)	62 (16.9)	437 (10.6)	71 (8.4)
	25**–**29	557 (24.8)	148 (40.3)	1,784 (43.4)	311 (36.7)
	30**–**34	732 (32.6)	94 (25.6)	1,315 (32.0)	342 (40.4)
	35**–**39	468 (20.8)	42 (11.4)	458 (11.1)	94 (11.1)
	≧40	282 (12.6)	16 (4.4)	108 (2.6)	23 (2.7)
	Missing	58 (2.6)	5 (1.4)	13 (0.3)	6 (0.7)
Father's education	Junior high school	125 (5.6)	25 (6.8)	191 (4.6)	35 (4.1)
	High school	767 (34.1)	161 (43.9)	1,463 (35.6)	288 (34.0)
	Vocational school	357 (15.9)	66 (18.0)	753 (18.3)	145 (17.1)
	Higher education	886 (39.4)	104 (28.3)	1,646 (40.0)	358 (42.3)
	Others	2 (0.1)	0 (0.0)	2 (0.1)	2 (0.2)
	Missing	110 (4.9)	11 (3.0)	60 (1.5)	19 (2.2)
Father's smoking status at age 0.5	No	849 (37.8)	110 (30.0)	1,690 (41.1)	337 (39.8)
	Yes	1,286 (57.2)	253 (68.9)	2,403 (58.4)	502 (59.3)
	Missing	112 (5.0)	4 (1.1)	22 (0.5)	8 (0.9)
Residential area	20 designated cities	647 (28.8)	70 (19.1)	865 (21.0)	220 (26.0)
	Other cities	1,265 (56.3)	204 (55.6)	2,475 (60.2)	495 (58.4)
	Rural	335 (14.9)	93 (25.3)	775 (18.8)	132 (15.6)

*SD, standard deviation.*

a *Overweight was determined as BMI z-score 1 SD or more according to the WHO criteria at the age of 8 years old (22).*

b *Rapid growth during infancy was determined as gaining more than 0.67 SD of weight from age 0.5 years to age 1.5 years (32).*

c *Excessive screen time was determined as 2 or more h of watching TV or playing computer games on either weekdays or weekends at the age of 8 years old (33).*

d *Unhealthy dinner habits were determined by frequent or occasional consumption of instant food and precooked food, using food delivery, or eating out on weekdays, which was asked at the age of 8 years old*.

**Table 3:** Characteristics of study participants from the Longitudinal Survey of Newborns in the 21st Century, 2001–2009 (girls *n* = 7,229).

		**Only children**	**Age gap shorter than 1.5 years**	**Age gap 1.5 years to shorter than 4 years**	**Age gap 4–8 years**
**Variable**		**No (%) or mean (SD)**	**No (%) or mean (SD)**	**No (%) or mean (SD)**	**No (%) or mean (SD)**
Number of participants		2,133 (29.5)	330 (4.6)	3,950 (54.6)	816 (11.3)
Overweight^a^	Yes	329 (15.4)	47 (14.2)	441 (11.2)	97 (11.9)
	No	1,804 (84.6)	283 (85.8)	3,509 (88.8)	719 (88.1)
Household income at age 0.5, million JPY	Mean, SD	3.9 (2.4)	3.6 (2.1)	3.8 (2.1)	3.9 (3.9)
Birth weight	<2,500 g	223 (10.5)	26 (7.9)	329 (8.3)	81 (9.9)
	≧2,500 g	1,910 (89.6)	304 (92.1)	3,621 (91.7)	735 (90.1)
Mother's age at birth, years	Mean, SD	30.7 (4.8)	27.9 (4.3)	28.4 (3.6)	28.5 (3.7)
	<25	241 (11.3)	80 (24.2)	647 (16.4)	133 (16.3)
	25**–**29	729 (34.2)	156 (47.3)	2,092 (53.0)	409 (50.1)
	30**–**34	744 (34.9)	74 (22.4)	1,054 (26.7)	238 (29.2)
	35**–**39	359 (16.8)	18 (5.5)	152 (3.9)	36 (4.4)
	≧s40	60 (2.8)	2 (0.6)	5 (0.1)	0 (0.0)
Mother's education	Junior high school	68 (3.2)	14 (4.2)	82 (2.1)	10 (1.2)
	High school	761 (35.7)	132 (40.0)	1,300 (32.9)	244 (29.9)
	Vocational school	853 (40.0)	139 (42.1)	1,821 (46.1)	414 (50.7)
	Higher education	400 (18.8)	39 (11.8)	696 (17.6)	144 (17.7)
	Others	2 (0.1)	2 (0.6)	3 (0.1)	0 (0.0)
	Missing	49 (2.3)	4 (1.2)	48 (1.2)	4 (0.5)
Mother's smoking status at age 0.5	No	1,792 (84.0)	274 (83.0)	3,465 (87.7)	696 (85.3)
	Yes	331 (15.5)	54 (16.4)	476 (12.1)	117 (14.3)
	Missing	10 (0.5)	2 (0.6)	9 (0.2)	3 (0.4)
Father residence at age 0.5	Yes	2,021 (94.8)	326 (98.8)	3,916 (99.1)	803 (98.4)
	No	112 (5.3)	4 (1.2)	34 (0.9)	13 (1.6)
Gestational period, weeks	22**–**36	94 (4.4)	11 (3.3)	102 (2.6)	22 (2.7)
	37**–**41	2,010 (94.2)	315 (95.5)	3,802 (96.3)	778 (95.3)
	≧42	29 (1.4)	4 (1.2)	46 (1.2)	16 (2.0)
Duration of breastfeeding	None	187 (8.8)	18 (5.5)	158 (4.0)	24 (2.9)
	1**–**2 months	462 (21.7)	103 (31.2)	564 (14.3)	141 (17.3)
	3**–**5 months	470 (22.0)	108 (32.7)	801 (20.3)	178 (21.8)
	6 or more months	999 (46.8)	98 (29.7)	2,382 (60.3)	460 (56.4)
	Missing	15 (0.7)	3 (0.9)	45 (1.1)	13 (1.6)
Rapid growth during early childhood^b^	No	1,096 (51.4)	161 (48.8)	2,157 (54.6)	460 (56.4)
	Yes	836 (39.2)	127 (38.5)	1,525 (38.6)	299 (36.6)
	Missing	201 (9.4)	42 (12.7)	268 (6.8)	57 (7.0)
Exercise habit at age 8	Yes	1,064 (49.9)	134 (40.6)	1,947 (49.3)	417 (51.1)
	No	1,069 (50.1)	196 (59.4)	2,003 (50.7)	399 (48.9)
Breakfast consumption at age 8	Yes	2,020 (94.7)	316 (95.8)	3,786 (95.9)	775 (95.0)
	No	59 (2.8)	11 (3.3)	94 (2.4)	29 (3.6)
	Missing	54 (2.5)	3 (0.9)	70 (1.8)	12 (1.5)
Excessive screen time at age 8^c^	No	673 (31.6)	120 (36.4)	1,389 (35.2)	231 (28.3)
	Yes	1,456 (68.3)	210 (63.6)	2,556 (64.7)	584 (71.6)
	Missing	4 (0.2)	0 (0.0)	5 (0.1)	1 (0.1)
Irregular bedtime at age 8	No	2,065 (96.8)	320 (97.0)	3,878 (98.2)	791 (96.9)
	Yes	64 (3.0)	10 (3.0)	68 (1.7)	24 (2.9)
	Missing	4 (0.2)	0 (0.0)	4 (0.1)	1 (0.1)
Healthy dinner habits at age 8^d^	Yes	611 (28.7)	104 (31.5)	1,353 (34.3)	283 (34.7)
	No	1,515 (71.0)	224 (67.9)	2,576 (65.2)	529 (64.8)
	Missing	7 (0.3)	2 (0.6)	21 (0.5)	4 (0.5)
Grandparent cohabitation at age 8	No	1,592 (74.6)	260 (78.8)	3,123 (79.1)	660 (80.9)
	Yes	541 (25.4)	70 (21.2)	827 (20.9)	156 (19.1)
BMI at birth	Mean, SD	12.5 (1.1)	12.5 (1.2)	12.5 (1.1)	12.5 (1.1)
	Unavailable	25 (1.2)	3 (0.9)	26 (0.7)	13 (1.6)
BMI at age 1.5	Mean, SD	16.1 (1.4)	16.2 (1.4)	16.1 (1.3)	16.1 (1.4)
	Unavailable	218 (10.2)	51 (15.5)	324 (8.2)	70 (8.6)
Father's age at first birth, years	Mean, SD	32.9 (6.0)	30.2 (4.7)	30.2 (4.7)	30.1 (4.4)
	<25	160 (7.5)	51 (15.5)	410 (10.4)	83 (10.2)
	25**–**29	527 (24.7)	130 (39.4)	1,667 (42.2)	339 (41.5)
	30**–**34	675 (31.7)	86 (26.1)	1,279 (32.4)	285 (34.9)
	35**–**39	458 (21.5)	43 (13.0)	449 (11.4)	91 (11.2)
	≧40	244 (11.4)	16 (4.9)	125 (3.2)	13 (1.6)
	Missing	69 (3.2)	4 (1.2)	20 (0.5)	5 (0.6)
Father's education	Junior high school	114 (5.3)	30 (9.1)	182 (4.6)	34 (4.2)
	High school	718 (33.7)	142 (43.0)	1,440 (36.5)	275 (33.7)
	Vocational school	345 (16.2)	55 (16.7)	689 (17.4)	151 (18.5)
	Higher education	845 (39.6)	96 (29.1)	1,579 (40.0)	350 (42.9)
	Others	2 (0.1)	0 (0.0)	1 (0.03)	1 (0.1)
	Missing	109 (5.1)	7 (2.1)	59 (1.5)	5 (0.6)
Father's smoking status at age 0.5	No	816 (38.3)	102 (30.9)	1,626 (41.2)	336 (41.2)
	Yes	1,226 (57.5)	224 (67.9)	2,295 (58.1)	467 (57.2)
	Missing	91 (4.3)	4 (1.2)	29 (0.7)	13 (1.6)
Residential area	20 designated cities	587 (27.5)	60 (18.2)	861 (21.8)	187 (22.9)
	Other cities	1,214 (56.9)	189 (57.3)	2,370 (60.0)	505 (61.9)
	Rural	332 (15.6)	81 (24.6)	719 (18.2)	124 (15.2)

*SD, standard deviation.*

a *Overweight was determined as BMI z-score 1 SD or more according to the WHO criteria at the age of 8 years old (22).*

b *Rapid growth during infancy was determined as gaining more than 0.67 SD of weight from age 0.5 years to age 1.5 years (32).*

c *Excessive screen time was determined as 2 or more h of watching TV or playing computer games on either weekdays or weekends at the age of 8 years old (33).*

d *Unhealthy dinner habits were determined by frequent or occasional consumption of instant food and precooked food, using food delivery, or eating out on weekdays, which was asked at the age of 8 years old*.

**Table 4:** Association of length of only-child period and overweight at age 8 among boys, using logistic regression (*n* = 7,576).

		**Crude**		**Model 1** ^**b**^		**Model 2** ^**c**^	
		**Odds ratio**	**95% CI**	**Odds ratio**	**95% CI**	**Odds ratio**	**95% CI**
Length of only child period	Only children (*n* = 2,247)	Ref	Ref	Ref	Ref	Ref	Ref
	Age gap shorter than 1.5 years (*n* = 367)	0.90	(0.69, 1.19)	0.93	(0.70, 1.23)	0.89	(0.67, 1.20)
	Age gap 1.5 years to shorter than 4 years (*n* = 4,115)	**0.76**	**(0.67, 0.86)**	**0.82**	**(0.71, 0.94)**	**0.83**	**(0.72, 0.96)**
	Age gap 4–8 years (*n* = 847)	**0.70**	**(0.57, 0.87)**	**0.75**	**(0.61, 0.93)**	**0.78**	**(0.62, 0.97)**
Household income at age 0.5^a^		–	–	0.99	(0.96, 1.02)	0.99	(0.96, 1.02)
Birth weight^a^		–	–	**1.06**	**(1.04, 1.07)**	**1.12**	**(1.10, 1.14)**
Mother's age at birth^a^		–	–	**1.19**	**(1.02, 1.38)**	**1.19**	**(1.02, 1.39)**
Mother's education level	Junior high school	–	–	Ref	Ref	Ref	Ref
	High school	–	–	0.74	(0.52, 1.04)	0.71	(0.50, 1.01)
	Vocational school	–	–	**0.69**	**(0.49, 0.97)**	**0.70**	**(0.49, 0.998)**
	Higher education	–	–	**0.58**	**(0.40, 0.84)**	**0.59**	**(0.40, 0.86)**
	Others	–	–	1.26	(0.32, 5.01)	2.07	(0.51, 8.36)
	Missing	–	–	0.90	(0.53, 1.53)	0.84	(0.48, 1.50)
Mother's smoking status at age 0.5	No	–	–	Ref	Ref	Ref	Ref
	Yes	–	–	**1.41**	**(1.19, 1.67)**	**1.36**	**(1.14, 1.63)**
	Missing	–	–	1.87	(0.85, 4.14)	1.79	(0.78, 4.09)
Father residence at age 0.5	Yes	–	–	Ref	Ref	Ref	Ref
	No	–	–	1.28	(0.89, 1.86)	1.18	(0.80, 1.72)
Preterm birth	No	–	–	–	–	Ref	Ref
	Yes	–	–	–	–	**1.38**	**(1.00, 1.89)**
Duration of breastfeeding	None	–	–	–	–	Ref	Ref
	1–2 months	–	–	–	–	1.02	(0.77, 1.35)
	3–5 months	–	–	–	–	0.89	(0.67, 1.18)
	6 or more months	–	–	–	–	1.05	(0.81, 1.36)
	Missing	–	–	–	–	1.16	(0.64, 2.08)
Rapid growth during early childhood^d^	No	–	–	–	–	Ref	Ref
	Yes	–	–	–	–	**3.07**	**(2.67, 3.52)**
	Missing	–	–	–	–	**1.79**	**(1.40, 2.29)**
Exercise habit at age 8	Yes	–	–	–	–	Ref	Ref
	No	–	–	–	–	1.12	(0.99, 1.27)
Breakfast consumption at age 8	Yes	–	–	–	–	Ref	Ref
	No	–	–	–	–	1.30	(0.94, 1.80)
	Missing	–	–	–	–	0.93	(0.58, 1.49)
Excessive screen time at age 8^e^	No	–	–	–	–	Ref	Ref
	Yes	–	–	–	–	**1.24**	**(1.08, 1.42)**
	Missing	–	–	–	–	1.00	(0.21, 4.85)
Irregular sleeping hours at age 8	No	–	–	–	–	Ref	Ref
	Yes	–	–	–	–	0.90	(0.60, 1.34)
	Missing	–	–	–	–	0.84	(0.18, 3.99)
Healthy dinner habits at age 8^f^	Yes	–	–	–	–	Ref	Ref
	No	–	–	–	–	1.06	(0.93, 1.21)
	Missing	–	–	–	–	0.32	(0.04, 2.56)
Grandparent cohabitation at age 8	No	–	–	–	–	Ref	Ref
	Yes	–	–	–	–	**1.23**	**(1.07, 1.41)**

*CI; confidence interval.*

*Bolded values indicate statistical significance at p <0.05.*

a *Mother's age at birth, birth weight and household income at age 0.5 were scaled so that the effect of a one-unit change can be interpreted as the effect of a 10-year change in age, 100-g change in weight, and one million JPY change in income, respectively.*

b *Model 1 adjusted for household income at age 0.5, birth weight, mother's age at birth, mother's education level, mother's smoking at age 0.5, and father cohabitation at age 0.5.*

c *Model 2 adjusted for preterm birth, duration of breastfeeding, rapid growth during early childhood, exercise habit at age 8, breakfast consumption at age 8, excessive screen time at age 8, irregular bedtime at age 8, healthy dinner habits at age 8, and grandparent residence at age 8 in addition to covariates adjusted in Model 1.*

d *Rapid growth during infancy was determined as gaining more than 0.67 SD of weight from age 0.5 years to age 1.5 years (32).*

e *Excessive screen time was determined as 2 or more h of watching TV or playing computer games on either weekdays or weekends at the age of 8 years old (33).*

f *Unhealthy dinner habits were determined by frequent or occasional consumption of instant food and precooked food, using food delivery, or eating out on weekdays, which was asked at the age of 8 years old*.

**Table 5:** Association of length of only-child period and overweight at age 8 among girls, using logistic regression (*n* = 7,229).

		**Crude**		**Model 1** ^**b**^		**Model 2** ^**c**^	
		**Odds ratio**	**95% CI**	**Odds ratio**	**95% CI**	**Odds ratio**	**95% CI**
Length of only child period	Only children (*n* =2,133)	Ref	Ref	Ref	Ref	Ref	Ref
	Age gap shorter than 1.5 years (*n* = 330)	0.91	(0.65, 1.27)	0.89	(0.64, 1.25)	0.91	(0.64, 1.29)
	Age gap 1.5 years to shorter than 4 years (*n* = 3,950)	**0.69**	**(0.59, 0.80)**	**0.73**	**(0.62, 0.86)**	**0.74**	**(0.62, 0.87)**
	Age gap 4–8 years (*n* = 816)	**0.74**	**(0.58, 0.94)**	0.79	(0.61, 1.01)	0.80	(0.62, 1.03)
Household income at age 0.5^a^		–	–	**0.94**	**(0.90, 0.98)**	**0.95**	**(0.92, 0.99)**
Birth weight^a^		–	–	**1.05**	**(1.03, 1.07)**	**1.13**	**(1.10, 1.15)**
Mother's age at birth^a^		–	–	**1.22**	**(1.02, 1.45)**	1.19	(0.99, 1.42)
Mother's education level	Junior high school	–	–	Ref	Ref	Ref	Ref
	High school	–	–	0.72	(0.49, 1.06)	0.76	(0.51, 1.14)
	Vocational school	–	–	**0.56**	**(0.38, 0.83)**	**0.61**	**(0.41, 0.93)**
	Higher education	–	–	**0.47**	**(0.30, 0.72)**	**0.51**	**(0.32, 0.80)**
	Others	–	–	1.81	(0.33, 10.02)	2.05	(0.36, 11.53)
	Missing	–	–	0.69	(0.36, 1.33)	0.65	(0.32, 1.31)
Mother's smoking status at age 0.5	No	–	–	Ref	Ref	Ref	Ref
	Yes	–	–	**1.34**	**(1.11, 1.62)**	**1.31**	**(1.07, 1.60)**
	Missing	–	–	1.47	(0.50, 4.35)	1.17	(0.39, 3.53)
Father residence at age 0.5	Yes	–	–	Ref	Ref	Ref	Ref
	No	–	–	1.13	(0.74, 1.71)	1.02	(0.66, 1.57)
Preterm birth	No	–	–	–	–	Ref	Ref
	Yes	–	–	–	–	**2.07**	**(1.40, 3.06)**
Duration of breastfeeding	None	–	–	–	–	Ref	Ref
	1–2 months	–	–	–	–	0.85	(0.62, 1.19)
	3–5 months	–	–	–	–	0.83	(0.60, 1.14)
	6 or more months	–	–	–	–	0.99	(0.73, 1.34)
	Missing	–	–	–	–	1.34	(0.68, 2.66)
Rapid growth during early childhood^d^	No	–	–	–	–	Ref	Ref
	Yes	–	–	–	–	**3.07**	**(2.59, 3.64)**
	Missing	–	–	–	–	**1.93**	**(1.45, 2.58)**
Exercise habit at age 8	Yes	–	–	–	–	Ref	Ref
	No	–	–	–	–	0.998	(0.86, 1.16)
Breakfast consumption at age 8	Yes	–	–	–	–	Ref	Ref
	No	–	–	–	–	**2.05**	**(1.43, 2.93)**
	Missing	–	–	–	–	0.89	(0.51, 1.55)
Excessive screen time at age 8^e^	No	–	–	–	–	Ref	Ref
	Yes	–	–	–	–	**1.35**	**(1.15, 1.59)**
	Missing	–	–	–	–	1.12	(0.14, 9.18)
Irregular sleeping hours at age 8	No	–	–	–	–	Ref	Ref
	Yes	–	–	–	–	1.17	(0.76, 1.81)
	Missing	–	–	–	–	1	–
Healthy dinner habits at age 8^f^	Yes	–	–	–	–	Ref	Ref
	No	–	–	–	–	**1.20**	**(1.02, 1.41)**
	Missing	–	–	–	–	**4.08**	**(1.66, 9.99)**
Grandparent cohabitation at age 8	No	–	–	–	–	Ref	Ref
	Yes	–	–	–	–	**1.36**	**(1.15, 1.60)**

*CI, confidence interval.*

*Bolded values indicate statistical significance at p < 0.05.*

a *Mother's age at birth, birth weight and household income at age 0.5 were scaled so that the effect of a one-unit change can be interpreted as the effect of a 10-year change in age, 100-g change in weight, and one million JPY change in income, respectively.*

b *Model 1 adjusted for household income at age 0.5, birth weight, mother's age at birth, mother's education level, mother's smoking at age 0.5, and father cohabitation at age 0.5.*

c *Model 2 adjusted for preterm birth, duration of breastfeeding, rapid growth during early childhood, exercise habit at age 8, breakfast consumption at age 8, excessive screen time at age 8, irregular bedtime at age 8, healthy dinner habits at age 8, and grandparent residence at age 8 in addition to covariates adjusted in Model 1.*

d *Rapid growth during infancy was determined as gaining more than 0.67 SD of weight from age 0.5 years to age 1.5 years (32).*

e *Excessive screen time was determined as 2 or more h of watching TV or playing computer games on either weekdays or weekends at the age of 8 years old (33).*

f *Unhealthy dinner habits were determined by frequent or occasional consumption of instant food and precooked food, using food delivery, or eating out on weekdays, which was asked at the age of 8 years old*.

After adjusting for other covariates (i.e., preterm birth, breastfeeding, rapid growth during early childhood, exercise habits, breakfast consumption, excessive screen time, irregular sleeping hours, bad dinner habits, and grandparent residence at the age of 8 years old) in addition to covariates adjusted in Model 1, boys who experienced birth intervals between 1.5 and 4 years were still less likely to be overweight (OR: 0.83, 95% CI, 0.72–0.96) (Table 4, Model 2). Moreover, boys who experienced birth intervals between 4 and 8 years remained less likely to be overweight (OR: 0.78, 95% CI, 0.62–0.97). Similarly, after adjusting for other covariates in addition to covariates adjusted in Model 1, among girls, those who experienced birth intervals between 1.5 and 4 years remained less likely to be overweight (OR: 0.75, 95% CI, 0.63–0.88) (Table 5, Model 2). The OR for girls who experienced birth intervals between 4 and 8 years was 0.80 (95% CI, 0.62–1.04), compared to an only child. Some covariates and other factors related to childhood overweight showed an association among boys, while others show an association among girls. Among both boys and girls, birth weight, mother's age at birth, mother's smoking status at age 0.5, preterm birth, rapid growth during early childhood, excessive screen time at age 8, and grandparent cohabitation were associated with childhood overweight, while mother's higher education level prevented childhood overweight. Among girls, in addition to the above, lack of breakfast consumption and unhealthy dinner habits were also associated with childhood overweight, while household income at the age of 0.5 prevented childhood overweight.

In sensitivity analysis, participants whose mother was under the age of 35 showed a similar trend as the original population in both boys and girls (Supplementary Tables A.1, A.2). We also conducted a sensitivity analysis to take into account the presence of overweight in early childhood and changes in BMI during early childhood. After adjusting for confounders and other covariates, similar results to the original analysis were obtained (Supplementary Tables A.3, A.4).

## Discussion

The results indicate that short birth intervals (<1.5 years) were not preventive for overweight in comparison with children who are the only child, but moderate birth intervals (between 1.5 and 4 years) were preventive for overweight risk. This is inconsistent with our hypothesis. Further, we have found that first-born children who experienced long birth intervals of 4–8 years during early childhood also had a lower risk of childhood overweight. To our knowledge, this is the first study to investigate the association of birth intervals experienced by the index child during early childhood and childhood overweight. Previous studies have shown that birth order is associated with overweight or obesity and that children with siblings are less likely to be overweight compared to those who are the only children (8–11). This research added to the literature that the risk of obesity in index children with siblings depends on the birth interval between the index child and the subsequent child.

When index children encountered moderate (1.5–4 years) birth intervals, index children were less likely to experience overweight in childhood, as hypothesized. However, when children faced long (4–8 years) birth intervals, which means being the only child up to the age of 4–8 years old, index children were less likely to experience overweight in childhood with the association being larger (i.e., more preventive). The lower risk was significant for both sexes when index children faced moderate (1.5–4 years) birth intervals. When the birth interval was long, a similarly lower risk was observed for both sexes, but the association did not reach statistical significance for girls. The association between moderate to long birth intervals and lower risk of overweight may be explained by the role of siblings as perceived by the index child. The index child believes that he/she should be a role model for their younger sibling in terms of having the correct behaviors, and may feel the responsibility to lead the subsequent child by example because parents expect the eldest child to take care of younger siblings (35). Thus, the index child might try to behave appropriately, especially in front of their younger sibling. When the age gap is larger, such role model behavior is more common and effective (36). This may lead to healthier behaviors and self-regulation that prevent children from becoming overweight among index children who experienced large age gaps.

As opposed to our hypothesis, short birth intervals (<1.5 years) in index children were not preventive for childhood overweight. This may be because they are less likely to share their food with their siblings as the index child and subsequent child can be raised with similar parental involvement (37). Another explanation may be the immature cognitive level of children under the age of 1.5 years old. The cognitive level from early infancy until the age of 2 years old is mainly focused on oneself and certain adults who hold the guardianship of the child (38), and not on their younger siblings. Thus, first-born children who experience a birth interval that is shorter than 1.5 years may not show such role model behaviors as their peers who experience moderate to long birth intervals.

This study has several limitations. First, height and weight were reported by the caregiver, and not measured objectively. However, the validity of caregiver-reported height and weight was reported in previous studies (19, 23). Second, there was insufficient information on the marital status, which may be a confounder for both having a second child and the BMI of the offspring through unfavorable nutrition due to the low socioeconomic status of single parenthood. Third, gestational diabetes was not available in the analysis due to no such question in the survey. This may be a confounder associated with birth intervals due to continuous treatment after pregnancy and with the BMI of the offspring through the concept of developmental origins of health and disease (DOHaD), explained through endocrine mechanisms (39).

This study implies that to prevent overweight in index children, the recommended birth interval for the subsequent child is 1.5 years or longer. However, overweight or weight gain is the “endpoint/phenomenon” of various factors. This study only focused on the interval/period of being only one child. There may be several reasons for the present phenomenon; further research should be conducted to validate the possible mechanism of stress due to family structure changes and mid-childhood overweight. In doing so, we believe that this study provided important basic data.

## Conclusion

This study found that having a second child between 1.5 and 8 years after the first birth prevents the first-born child from being overweight at the age of 8 years old compared to being the only child. Birth intervals shorter than 1.5 years have an overweight risk which is similar to that of only children. Further research to reveal the mechanism of this association is needed.

## Data Availability Statement

The original contributions presented in the study are included in the article/Supplementary Material, further inquiries can be directed to the corresponding author/s.

## Ethics Statement

The studies involving human participants were reviewed and approved by Ethics Committee of Tokyo Medical and Dental University. Written informed consent to participate in this study was provided by the participants' legal guardian/next of kin. The study was conducted according to the guidelines of the Declaration of Helsinki.

## Author Contributions

AK, NN, and TF: conceptualization. AK and NN: methodology and data curation. AK: software, formal analysis, investigation, writing—original draft preparation, and visualization. NN: validation. TF: resources and supervision. NN and TF: writing—review and editing. All authors contributed to the article and approved the submitted version.

## Conflict of Interest

The authors declare that the research was conducted in the absence of any commercial or financial relationships that could be construed as a potential conflict of interest.

## Publisher's Note

All claims expressed in this article are solely those of the authors and do not necessarily represent those of their affiliated organizations, or those of the publisher, the editors and the reviewers. Any product that may be evaluated in this article, or claim that may be made by its manufacturer, is not guaranteed or endorsed by the publisher.
