# Supplementary material for: Association Between Length of Only-Child Period During Early Childhood and Overweight at Age 8—A Population-Based Longitudinal Study in Japan
Source: Front Pediatr. 2022 Jun 14;10:782940. doi: 10.3389/fped.2022.782940 (PMC9237356; doi:10.3389/fped.2022.782940)
Supplement: Supplementary file 1 [file Table_1.pdf]

Table A.1: Association of length of only child period and overweight at age 8 among boys whose mother was under the age of 35 at birth, using logistic regression (n=6,900)

Table A.1. Association of length of only child period and overweight at age 8 among boys whose mother was under the age of 35 at birth, using logistic regression (n=6,900)

		Crude		Model 1 [b]		Model 2 [c]	
		Odds Ratio	95% CI	Odds Ratio	95% CI	Odds Ratio	95% CI
Length of only child period	Only children (n=1,781)	Ref	Ref	Ref	Ref	Ref	Ref
	Age gap shorter than 1.5 years (n=350)	0.92	(0.69, 1.22)	0.91	(0.68, 1.22)	0.88	(0.65, 1.19)
	Age gap 1.5 years to shorter than 4 years (n=3,950)	**0.76**	**(0.66, 0.87)**	**0.78**	**(0.68, 0.91)**	**0.79**	**(0.68, 0.92)**
	Age gap 4 -8 years (n=819)	**0.68**	**(0.55, 0.85)**	**0.71**	**(0.56, 0.88)**	**0.72**	**(0.57, 0.90)**
Household income at age 0.5 [a]		-	-	0.98	(0.95, 1.01)	0.99	(0.95, 1.02)
Birth weight [a]		-	-	**1.06**	**(1.04, 1.08)**	**1.12**	**(1.10, 1.14)**
Mother's age at birth [a]		-	-	1.17	(0.97, 1.42)	1.20	(0.98, 1.46)
Mother's education level	Junior high school	-	-	Ref	Ref	Ref	Ref
	High school	-	-	0.71	(0.50, 1.01)	**0.68**	**(0.48, 0.98)**
	Vocational school	-	-	**0.70**	**(0.49, 0.99)**	0.71	(0.49, 1.02)
	Higher education	-	-	**0.58**	**(0.39, 0.85)**	**0.59**	**(0.39, 0.88)**
	Others	-	-	1.23	(0.31, 4.91)	2.03	(0.50, 8.28)
	Missing	-	-	0.94	(0.54, 1.63)	0.90	(0.49, 1.62)
Mother's smoking status at age 0.5	No	-	-	Ref	Ref	Ref	Ref
	Yes	-	-	**1.39**	**(1.17, 1.66)**	**1.35**	**(1.12, 1.62)**

	Missing	-	-	1.51	(0.63, 3.63)	1.43	(0.57, 3.57)
Father residence at age 0.5	Yes	-	-	Ref	Ref	Ref	Ref
	No	-	-	1.01	(0.67, 1.54)	0.92	(0.61, 1.42)
Preterm birth	No	-	-	-	-	Ref	Ref
	Yes	-	-	-	-	1.25	(0.88, 1.78)
Duration of breastfeeding	None	-	-	-	-	Ref	Ref
	1-2 months	-	-	-	-	0.94	(0.70, 1.27)
	3-5months	-	-	-	-	0.83	(0.61, 1.12)
	6 or more months	-	-	-	-	1.00	(0.76, 1.33)
	Missing	-	-	-	-	1.13	(0.62, 2.08)
Rapid growth during early childhood [d]	No	-	-	-	-	Ref	Ref
	Yes	-	-	-	-	**3.10**	**(2.68, 3.59)**
	Missing	-	-	-	-	**1.74**	**(1.34, 2.24)**
Exercise habit at age 8	Yes	-	-	-	-	Ref	Ref
	No	-	-	-	-	1.13	(0.99, 1.29)
Breakfast consumption at age 8	Yes	-	-	-	-	Ref	Ref
	No	-	-	-	-	1.36	(0.96, 1.91)
	Missing	-	-	-	-	0.97	(0.60, 1.59)
Excessive screen time at age 8 [e]	No	-	-	-	-	Ref	Ref
	Yes	-	-	-	-	**1.28**	**(1.11, 1.48)**
	Missing	-	-	-	-	1.74	(0.33, 9.32)
Irregular sleeping hours at age 8	No	-	-	-	-	Ref	Ref
	Yes	-	-	-	-	0.88	(0.56, 1.37)

	Missing	-	-	-	-	0.39	(0.05, 3.17)
Healthy dinner habits at age 8 [f]	Yes	-	-	-	-	Ref	Ref
	No	-	-	-	-	1.05	(0.91, 1.21)
	Missing	-	-	-	-	1.00	-
Grandparent cohabitation at age 8	No	-	-	-	-	Ref	Ref
	Yes	-	-	-	-	**1.22**	**(1.06, 1.42)**

CI; confidence interval

Bolded values indicate statistical significance at p<0.05.

[a] Mother's age at birth, birth weight and household income at age 0.5 were scaled so that the effect of a one-unit change can be interpreted as the effect of a ten-year change in age, 100-gram change in weight, and one million JPY change in income, respectively.

[b] Model1 adjusted for household income at age 0.5, birth weight, mother's age at birth, mother's education level, mother's smoking at age 0.5 and father cohabitation at age 0.5.

[c] Model2 adjusted for preterm birth, duration of breastfeeding, rapid growth during early childhood, exercise habit at age 8, breakfast consumption at age 8, excessive screen time at age8, irregular bedtime at age 8, healthy dinner habits at age 8, and grandparent residence at age 8 in addition to covariates adjusted in Model 1.

[d] Rapid growth during infancy was determined as gaining more than 0.67SD of weight from age 0.5 years to age 1.5 years[32].

[e] Excessive screen time was determined as two or more hours of watching TV or playing computer games on either weekdays or weekends at the age of 8 years old[33].

[f] Unhealthy dinner habits were determined by frequent or occasional consumption of instant food and pre-cooked food, using food delivery, or eating out on weekdays, which was asked at the age of 8 years old.

Table A.2: Association of length of only child period and overweight at age 8 among girls whose mother was under the age of 35 at birth, using logistic regression (n=6,597)

Table A.2. Association of length of only child period and overweight at age 8 among girls whose mother was under the age of 35 at birth, using logistic regression (n=6,597)

		Crude		Model 1 [b]		Model 2 [c]	
		Odds Ratio	95% CI	Odds Ratio	95% CI	Odds Ratio	95% CI
Length of only child period	Only children (n=1,714)	Ref	Ref	Ref	Ref	Ref	Ref
	Age gap shorter than 1.5 years (n=310)	0.82	(0.57, 1.17)	0.78	(0.54, 1.12)	0.80	(0.55, 1.16)
	Age gap 1.5 years to shorter than 4 years (n=3,793)	**0.68**	**(0.58, 0.81)**	**0.70**	**(0.59, 0.84)**	**0.72**	**(0.61, 0.86)**
	Age gap 4 -8 years (n=780)	**0.75**	**(0.58, 0.97)**	0.78	(0.60, 1.004)	0.79	(0.60, 1.03)
Household income at age 0.5 [a]		-	-	**0.94**	**(0.90, 0.98)**	**0.95**	**(0.91, 0.99)**
Birth weight [a]		-	-	**1.06**	**(1.04, 1.08)**	**1.13**	**(1.11, 1.16)**
Mother's age at birth [a]		-	-	1.08	(0.86, 1.36)	1.09	(0.86, 1.37)
Mother's education level	Junior high school	-	-	Ref	Ref	Ref	Ref
	High school	-	-	0.82	(0.54, 1.24)	0.85	(0.55, 1.30)
	Vocational school	-	-	**0.63**	**(0.41, 0.96)**	0.68	(0.44, 1.05)
	Higher education	-	-	**0.50**	**(0.32, 0.80)**	**0.54**	**(0.33, 0.87)**
	Others	-	-	3.14	(0.54, 18.44)	3.10	(0.51, 18.84)
	Missing	-	-	0.78	(0.40, 1.55)	0.72	(0.35, 1.51)
Mother's smoking status at age 0.5	No	-	-	Ref	Ref	Ref	Ref
	Yes	-	-	1.32	(1.08, 1.61)	1.28	(1.04, 1.58)

	Missing	-	-	1.16	(0.34, 3.95)	0.91	(0.26, 3.16)
Father residence at age 0.5	Yes	-	-	Ref	Ref	Ref	Ref
	No	-	-	1.09	(0.68, 1.73)	0.97	(0.60, 1.56)
Preterm birth	No	-	-	-	-	Ref	Ref
	Yes	-	-	-	-	**1.86**	**(1.20, 2.87)**
Duration of breastfeeding	None	-	-	-	-	Ref	Ref
	1-2 months	-	-	-	-	0.90	(0.63, 1.28)
	3-5months	-	-	-	-	0.85	(0.60, 1.21)
	6 or more months	-	-	-	-	0.97	(0.70, 1.35)
	Missing	-	-	-	-	1.38	(0.67, 2.84)
Rapid growth during early childhood [d]	No	-	-	-	-	Ref	Ref
	Yes	-	-	-	-	**3.02**	**(2.52, 3.61)**
	Missing	-	-	-	-	**1.96**	**(1.45, 2.65)**
Exercise habit at age 8	Yes	-	-	-	-	Ref	Ref
	No	-	-	-	-	0.97	(0.83, 1.13)
Breakfast consumption at age 8	Yes	-	-	-	-	Ref	Ref
	No	-	-	-	-	**2.16**	**(1.49, 3.13)**
	Missing	-	-	-	-	0.93	(0.50, 1.76)
Excessive screen time at age 8 [e]	No	-	-	-	-	Ref	Ref
	Yes	-	-	-	-	**1.36**	**(1.14, 1.62)**
	Missing	-	-	-	-	1.80	(0.21, 15.2)
Irregular sleeping hours at age 8	No	-	-	-	-	Ref	Ref
	Yes	-	-	-	-	1.19	(0.77, 1.85)

	Missing	-	-	-	-	1.00	-
Healthy dinner habits at age 8 [f]	Yes	-	-	-	-	Ref	Ref
	No	-	-	-	-	**1.21**	**(1.02, 1.43)**
	Missing	-	-	-	-	**3.50**	**(1.25, 9.84)**
Grandparent cohabitation at age 8	No	-	-	-	-	Ref	Ref
	Yes	-	-	-	-	**1.30**	**(1.09, 1.55)**

CI; confidence interval

Bolded values indicate statistical significance at $p<0.05$.

[a] Mother's age at birth, birth weight and household income at age 0.5 were scaled so that the effect of a one-unit change can be interpreted as the effect of a ten-year change in age, 100-gram change in weight, and one million JPY change in income, respectively.

[b] Model1 adjusted for household income at age 0.5, birth weight, mother's age at birth, mother's education level, mother's smoking at age 0.5 and father cohabitation at age 0.5.

[c] Model2 adjusted for preterm birth, duration of breastfeeding, rapid growth during early childhood, exercise habit at age 8, breakfast consumption at age 8, excessive screen time at age8, irregular bedtime at age 8, healthy dinner habits at age 8, and grandparent residence at age 8 in addition to covariates adjusted in Model 1.

[d] Rapid growth during infancy was determined as gaining more than 0.67SD of weight from age 0.5 years to age 1.5 years[32].

[e] Excessive screen time was determined as two or more hours of watching TV or playing computer games on either weekdays or weekends at the age of 8 years old[33].

[f] Unhealthy dinner habits were determined by frequent or occasional consumption of instant food and pre-cooked food, using food delivery, or eating out on weekdays, which was asked at the age of 8 years old.

Table A.3: Association of length of only child period and overweight at age 8 among boys, using logistic regression (n=6,779)

Table A.3. Association of length of only child period and overweight at age 8 among boys, using logistic regression (n=6,779)

		Crude		Model 1 [b]		Model 2 [c]	
		Odds Ratio	95% CI	Odds Ratio	95% CI	Odds Ratio	95% CI
Length of only child period	Only children (n=2,003)	Ref	Ref	Ref	Ref	Ref	Ref
	Age gap shorter than 1.5 years (n=312)	0.90	(0.67, 1.21)	0.92	(0.68, 1.25)	0.87	(0.64, 1.20)
	Age gap 1.5 years to shorter than 4 years (n=3,707)	**0.74**	**(0.65, 0.85)**	**0.80**	**(0.69, 0.93)**	**0.81**	**(0.69, 0.94)**
	Age gap 4 -8 years (n=757)	**0.66**	**(0.53, 0.83)**	**0.70**	**(0.56, 0.88)**	**0.75**	**(0.59, 0.95)**
Household income at age 0.5 [a]		-	-	0.99	(0.97, 1.02)	0.99	(0.96, 1.02)
Birth weight [a]		-	-	**1.07**	**(1.05, 1.08)**	**1.08**	**(1.05, 1.11)**
Mother's age at birth [a]		-	-	1.16	(0.99, 1.36)	1.17	(0.99, 1.38)
Mother's education level	Junior high school	-	-	Ref	Ref	Ref	Ref
	High school	-	-	0.82	(0.56, 1.20)	0.79	(0.53, 1.17)
	Vocational school	-	-	0.77	(0.53, 1.13)	0.79	(0.53, 1.17)
	Higher education	-	-	0.68	(0.45, 1.02)	0.68	(0.44, 1.04)
	Others	-	-	1.42	(0.35, 5.72)	2.37	(0.56, 9.98)
	Missing	-	-	0.62	(0.13, 3.02)	0.76	(0.15, 3.77)
Mother's smoking at age 0.5	No	-	-	Ref	Ref	Ref	Ref
	Yes	-	-	**1.52**	**(1.27, 1.82)**	**1.39**	**(1.15, 1.69)**
	Missing	-	-	1.42	(0.56, 3.61)	1.32	(0.49, 3.57)
Father residence at age 0.5	Yes	-	-	Ref	Ref	Ref	Ref

	No	-	-	1.32	(0.88, 1.97)	1.20	(0.80, 1.82)
BMI SD at birth		-	-	-	-	1.50	(1.32, 1.70)
Change in BMI SD from birth to age 1.5		-	-	-	-	1.42	(1.31, 1.53)
Preterm birth	No	-	-	-	-	Ref	Ref
	Yes	-	-	-	-	1.06	(0.74, 1.52)
Duration of breastfeeding	None	-	-	-	-	Ref	Ref
	1-2 months	-	-	-	-	0.91	(0.67, 1.22)
	3-5months	-	-	-	-	0.80	(0.59, 1.08)
	6 or more months	-	-	-	-	0.92	(0.69, 1.21)
	Missing	-	-	-	-	0.81	(0.41, 1.60)
Rapid growth during early childhood [d]	No	-	-	-	-	Ref	Ref
	Yes	-	-	-	-	**2.22**	**(1.88, 2.62)**
	Missing	-	-	-	-	1.00	-
Exercise habit at age 8	Yes	-	-	-	-	Ref	Ref
	No	-	-	-	-	1.08	(0.94, 1.24)
Breakfast consumption at age 8	Yes	-	-	-	-	Ref	Ref
	No	-	-	-	-	1.27	(0.89, 1.82)
	Missing	-	-	-	-	0.87	(0.51, 1.48)
Excessive screen time at age 8 [e]	No	-	-	-	-	Ref	Ref
	Yes	-	-	-	-	**1.25**	**(1.08, 1.45)**
	Missing	-	-	-	-	1.02	(0.20, 5.24)
Irregular sleeping hours at age 8	No	-	-	-	-	Ref	Ref
	Yes	-	-	-	-	0.98	(0.64, 1.49)

	Missing	-	-	-	-	0.96	(0.19, 4.86)
Healthy dinner habits at age 8 [f]	Yes	-	-	-	-	Ref	Ref
	No	-	-	-	-	1.08	(0.94, 1.25)
	Missing	-	-	-	-	1.00	-
Grandparent cohabitation at age 8	No	-	-	-	-	Ref	Ref
	Yes	-	-	-	-	1.16	(0.995, 1.34)

CI; confidence interval

SD; standard deviation

Bolded values indicate statistical significance at $p<0.05$.

[a] Mother's age at birth, birth weight and household income at age 0.5 were scaled so that the effect of a one-unit change can be interpreted as the effect of a ten-year change in age, 100-gram change in weight, and one million JPY change in income, respectively.

[b] Model1 adjusted for household income at age 0.5, birth weight, mother's age at birth, mother's education level, mother's smoking at age 0.5 and father cohabitation at age 0.5.

[c] Model2 adjusted for preterm birth, duration of breastfeeding, rapid growth during early childhood, exercise habit at age 8, breakfast consumption at age 8, excessive screen time at age8, irregular bedtime at age 8, healthy dinner habits at age 8, and grandparent residence at age 8 in addition to covariates adjusted in Model 1.

[d] Rapid growth during infancy was determined as gaining more than 0.67SD of weight from age 0.5 years to age 1.5 years[32].

[e] Excessive screen time was determined as two or more hours of watching TV or playing computer games on either weekdays or weekends at the age of 8 years old[33].

[f] Unhealthy dinner habits were determined by frequent or occasional consumption of instant food and pre-cooked food, using food delivery, or eating out on weekdays, which was asked at the age of 8 years old.

Table A.4: Association of length of only child period and overweight at age 8 among girls, using logistic regression (n=6,507)

Table A.4. Association of length of only child period and overweight at age 8 among girls, using logistic regression (n=6,507)

		Crude		Model 1 [b]		Model 2 [c]	
		Odds Ratio	95% CI	Odds Ratio	95% CI	Odds Ratio	95% CI
Length of only child period	Only children (n=1,893)	Ref	Ref	Ref	Ref	Ref	Ref
	Age gap shorter than 1.5 years (n=277)	0.89	(0.62, 1.28)	0.85	(0.59, 1.24)	0.85	(0.58, 1.25)
	Age gap 1.5 years to shorter than 4 years (n=3,603)	**0.66**	**(0.56, 0.78)**	**0.69**	**(0.58, 0.82)**	**0.67**	**(0.56, 0.81)**
	Age gap 4 -8 years (n=734)	**0.77**	**(0.60, 0.99)**	0.80	(0.62, 1.04)	0.78	(0.59, 1.02)
Household income at age 0.5 [a]		-	-	**0.95**	**(0.91, 0.99)**	0.96	(0.92, 1.00)
Birth weight [a]		-	-	**1.05**	**(1.03, 1.07)**	**1.07**	**(1.04, 1.11)**
Mother's age at birth [a]		-	-	1.18	(0.98, 1.43)	1.15	(0.94, 1.39)
Mother's education level	Junior high school	-	-	Ref	Ref	Ref	Ref
	High school	-	-	0.82	(0.53, 1.27)	0.85	(0.53, 1.35)
	Vocational school	-	-	0.66	(0.42, 1.02)	0.70	(0.44, 1.12)
	Higher education	-	-	0.54	(0.33, 0.87)	**0.55**	**(0.33, 0.91)**
	Others	-	-	2.11	(0.38, 11.85)	2.59	(0.42, 15.88)
	Missing	-	-	1.11	(0.12, 10.69)	1.37	(0.13, 14.25)
Mother's smoking at age 0.5	No	-	-	Ref	Ref	Ref	Ref
	Yes	-	-	**1.29**	**(1.05, 1.59)**	**1.28**	**(1.02, 1.59)**
	Missing	-	-	0.83	(0.19, 3.61)	0.57	(0.13, 2.51)
Father residence at age 0.5	Yes	-	-	Ref	Ref	Ref	Ref

	No	-	-	1.13	(0.72, 1.77)	0.93	(0.58, 1.51)
BMI SD at birth		-	-	-	-	**1.69**	**(1.46, 1.96)**
Change in BMI SD from birth to age 1.5		-	-	-	-	**1.62**	**(1.47, 1.79)**
Preterm birth	No	-	-	-	-	Ref	Ref
	Yes	-	-	-	-	**1.81**	**(1.19, 2.78)**
Duration of breastfeeding	None	-	-	-	-	Ref	Ref
	1-2 months	-	-	-	-	0.87	(0.61, 1.25)
	3-5months	-	-	-	-	0.87	(0.61, 1.24)
	6 or more months	-	-	-	-	1.03	(0.74, 1.44)
	Missing	-	-	-	-	1.80	(0.89, 3.64)
Rapid growth during early childhood [d]	No	-	-	-	-	Ref	Ref
	Yes	-	-	-	-	**1.95**	**(1.59, 2.38)**
	Missing	-	-	-	-	1.00	-
Exercise habit at age 8	Yes	-	-	-	-	Ref	Ref
	No	-	-	-	-	0.96	(0.82, 1.12)
Breakfast consumption at age 8	Yes	-	-	-	-	Ref	Ref
	No	-	-	-	-	**2.03**	**(1.37, 3.02)**
	Missing	-	-	-	-	0.99	(0.55, 1.79)
Excessive screen time at age 8 [e]	No	-	-	-	-	Ref	Ref
	Yes	-	-	-	-	**1.31**	**(1.10, 1.56)**
	Missing	-	-	-	-	0.74	(0.09, 6.27)
Irregular sleeping hours at age 8	No	-	-	-	-	Ref	Ref
	Yes	-	-	-	-	1.00	(0.62, 1.63)

	Missing	-	-	-	-	1.00	-
Healthy dinner habits at age 8 [f]	Yes	-	-	-	-	Ref	Ref
	No	-	-	-	-	**1.21**	**(1.02, 1.43)**
	Missing	-	-	-	-	**3.70**	**(1.32, 10.35)**
Grandparent cohabitation at age 8	No	-	-	-	-	Ref	Ref
	Yes	-	-	-	-	**1.42**	**(1.19, 1.69)**

CI; confidence interval

SD; standard deviation

Bolded values indicate statistical significance at $p<0.05$.

[a] Mother's age at birth, birth weight and household income at age 0.5 were scaled so that the effect of a one-unit change can be interpreted as the effect of a ten-year change in age, 100-gram change in weight, and one million JPY change in income, respectively.

[b] Model1 adjusted for household income at age 0.5, birth weight, mother's age at birth, mother's education level, mother's smoking at age 0.5 and father cohabitation at age 0.5.

[c] Model2 adjusted for preterm birth, duration of breastfeeding, rapid growth during early childhood, exercise habit at age 8, breakfast consumption at age 8, excessive screen time at age8, irregular bedtime at age 8, healthy dinner habits at age 8, and grandparent residence at age 8 in addition to covariates adjusted in Model 1.

[d] Rapid growth during infancy was determined as gaining more than 0.67SD of weight from age 0.5 years to age 1.5 years[32].

[e] Excessive screen time was determined as two or more hours of watching TV or playing computer games on either weekdays or weekends at the age of 8 years old[33].

[f] Unhealthy dinner habits were determined by frequent or occasional consumption of instant food and pre-cooked food, using food delivery, or eating out on weekdays, which was asked at the age of 8 years old.
